# Alpha Glucosidase Inhibitory Activities of Plants with Focus on Common Vegetables

**DOI:** 10.3390/plants9010002

**Published:** 2019-12-18

**Authors:** Samuel Tilahun Assefa, Eun-Young Yang, Soo-Young Chae, Mihye Song, Jundae Lee, Myeong-Cheoul Cho, Seonghoe Jang

**Affiliations:** 1 National Institute of Horticultural and Herbal Science (NIHHS), Rural Development Administration (RDA), Wanju-gun, Jellabuk-do 55365, Korea; sumalew70@gmail.com (S.T.A.); yangyang2@korea.kr (E.-Y.Y.); cotez@korea.kr (S.-Y.C.); chomc@korea.kr (M.-C.C.); 2 Department of Horticulture, College of Agriculture and Life Sciences, Jeonbuk National University, Jeonju-si, Jeollabuk-do 54896, Korea; ajfall@jbnu.ac.kr; 3 World Vegetable Center Korea Office (WKO), Wanju-gun, Jellabuk-do 55365, Korea; mihye.song@worldveg.org

**Keywords:** alpha-glucosidase, alpha-glucosidase inhibitor, breeding, diabetes, secondary metabolites, vegetables

## Abstract

Type-2 diabetes mellitus is one of the most prevalent metabolic diseases in the world, and is characterized by hyperglycemia (i.e., high levels of glucose in the blood). Alpha-glucosidases are enzymes in the digestive tract that hydrolyze carbohydrates into glucose. One strategy that has been developed to treat type-2 diabetes is inhibition of the activity of alpha-glucosidases using synthetic drugs. However, these inhibitors are usually associated with gastrointestinal side effects. Therefore, the development of inhibitors from natural products offers an alternative option for the control of hyperglycemia. In recent years, various studies have been conducted to identify alpha-glucosidases inhibitors from natural sources such as plants, and many candidates have transpired to be secondary metabolites including alkaloids, flavonoids, phenols, and terpenoids. In this review, we focus on the alpha-glucosidases inhibitors found in common vegetable crops and the major classes of phytochemicals responsible for the inhibitory activity, and also as potential/natural drug candidates for the treatment of type-2 diabetes mellitus. In addition, possible breeding strategies for production of improved vegetable crops with higher content of the inhibitors are also described.

## 1. Introduction

**Figure 1:**
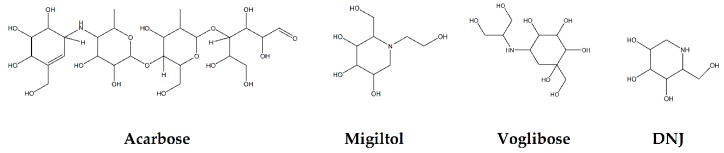
Molecular structure of representative commercialized glucosidase inhibitor drugs.

Glycosidases catalyzing the hydrolysis of glycosidic bonds in polysaccharides and glycoconjugates, play critical roles in various biological processes, including carbohydrate digestion, lysosomal catabolism of glycoconjugates, and post-translational modifications of cellular glycoproteins [1,2]. In particular, mammalian α-glucosidase (AG) in the mucosal brush border of the small intestine catalyzes the end step of digestion of starch and disaccharides that are abundant in the human diet. Inhibitors of AG delay the breakdown of carbohydrates in the small intestine and diminish the postprandial blood glucose excursion; thus, inhibition of glycosidases has a significant effect on polysaccharide metabolism, glycoprotein processing, and cellular interaction, widening opportunities for the discovery and development of new therapeutic agents against diseases such as diabetes, obesity, metastatic cancer, and viral infection [3,4]. In particular, AG as a glucosidase located in the brush border of the small intestine is able to selectively hydrolyze terminal (1→4)-linked α-glucose residues (starch or disaccharides) to release a single α-glucose molecule [5,6]. Therefore, various types of potential α-glucosidase inhibitors (AGIs) have been extensively screened or studied and acarbose, miglitol, voglibose, and 1-deoxynojirimycin (DNJ) are currently commercialized anti-glucosidase drugs (Figure 1; [7]) against type-2 diabetes, a chronic condition in which the body becomes resistant to the normal effects of insulin, resulting in ineffectiveness at managing the blood glucose levels.

**Figure 2:**
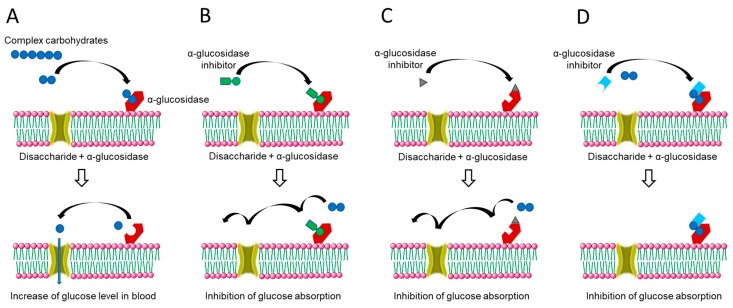
Basic modes of action of α-glucosidase inhibitors (AGIs). (**A**) Absorption of glucose produced from carbohydrates by hydrolytic glucosidase activity of α-glucosidase (AG) in the small intestine. (**B**–**D**) Competitive, non-competitive (allosteric), and uncompetitive inhibition of the AG activity by AGIs, respectively.

Most AGIs can attach to the carbohydrate binding site of AG due to their similarity with disaccharides or oligosaccharides in molecular structure. Moreover, the complexes have a stronger affinity than the carbohydrate–glucosidase complexes (Figure 2; [8]). In addition, non-competitive, uncompetitive or mixed types of inhibition of AG activities from some flavonoid-based compounds have been reported (Figure 2; [9]). Thus, inhibition of enzymes involved in the digestion of carbohydrates is able to significantly decrease the postprandial increase of glucose level in the blood after a mixed carbohydrate diet, which has been shown to be essential in preventing the progress of impaired glucose tolerance towards type-2 diabetes [10].

Although the activity of AG in the mucous membrane of the small intestine can be inhibited by commercial AGIs, these pharmaceutical drugs formulated for the inhibition of the key enzymes frequently come with attendant side effects and expensive cost [5,11]. Thus, an economical alternative to managing the disease with few or no side effects is an attractive option. Recent studies on the beneficial health effects of vegetables have piqued the interest of researchers due to their possible protection against chronic diseases. In addition, continuous effort is being made in the research and discovery of new anti-glucosidase/anti-diabetic drugs with higher safety profiles for long-term therapy [12,13,14]. Thus, it is worth reviewing the effects of common vegetables for the purpose of identifying novel/potential compounds that may be suitable for development as anti-diabetes agents. In this review, we will summarize research results on the AGI activity in plants, with emphasis on common vegetables, and also briefly describe possible means of producing vegetables harboring higher levels of AGI activity in the future.

## 2. Natural Compounds as Potential Candidates for AGIs

A range of chemical compounds identified from various plants shows inhibitory activities against AG enzymes. Most AGIs are secondary metabolites such as alkaloids, phenolic acids, flavonoids, terpenoids, anthocyanins, and their glycosides (Table 1). Alkaloids are mainly found in certain flowering plants [15]. Species from the Berberidaceae family are outstanding alkaloid-yielding plants. However, alkaloids can also be obtained from other sources such as animals, bacteria, and fungi. Plant-derived alkaloids are known to be repellents that protect plants against insects and herbivores. Phenolic compounds are secondary metabolites found most abundantly in plants. Phenolic acids are polyphenols containing the C6 aromatic ring of hydroxybenzoic acids including gallic acid, caffeic acid, and coumaric acid. The synthesis of phenolic compounds in plants is promoted by biotic and abiotic stresses (i.e., herbivores, pathogens, saline stress, heavy metal stress, unfavorable temperature, pH, and UV radiation) [16]. Flavonoids are also polyphenols which present in distinct tissues and organs in various plant species. It has been reported that flavonoids help plants to protect against adverse environmental constraints and also contribute to the growth and development of plants [17]. Terpenes are the most diverse natural products and are formed by a linear arrangement of a single building block called isoprene, also known as 2-methylbuta-1, 3-diene (C5H8). Plants employ terpenoid metabolites for a variety of fundamental functions in growth and development and also use the majority of terpenoids for their protection in the abiotic and biotic environment [18]. From these classes of compounds, a number of individual compounds are reported to show AGI activity because of the special structure or functional groups they possess (Table 1). In addition to the use of secondary metabolites, efforts to explore and develop peptide-based anti-diabetic agents against mammalian intestinal AG are underway [19]. Although the natural substrates of glycosidase are polysaccharides, peptide modulators of AG may have huge potential based on structural features of AGIs with the characteristic sugar-mimetic structure [19,20]. Furthermore, their high affinity and specificity in interactions with the protein targets, and reduced immunogenicity and low toxicity profiles, in general, would be additional benefits of peptide-based AGIs [20]. Iminosugars are another class of compounds that inhibit carbohydrate hydrolyzing enzymes [21,22]. These are sometimes called sugar-shaped alkaloids, polyhydroxy alkaloids, azasugars, or aminosugars due to their structural similarity with sugars [23]. Nojirimycin and fagomine were the first natural iminosugars to be discovered from microbe (*Streptomyces*) and plant (*Fagopyrum esculentum*). Iminosugars play an important role in chemotaxonomy and exhibit antimicrobial properties. Moreover, nojirimycin showed potent AG inhibitory activity, which could be due to its structural resemblance to glucose.

## 3. Plants with AG Inhibitory Activity

Plants are the major source of phytochemicals, and some of these compounds possess different health promoting functions. Various types of these compounds are being isolated from different plant species and studied for their potential to manage type-2 diabetes, and extracts of leaves, roots, barks, and fruits from different medicinal plants, herbs, and other plants are reported to exhibit inhibitory activity against AG [41,42]. A 13-membered ring thiocyclitol (13-MRT) compound isolated from the medicinal tree, *Salacia reticulate*, used as an antidiabetic, was reported to show potential AGI activity with IC_50_ values of 0.23 and 0.19 µM against maltase and sucrase enzymes, respectively [43]. *Morus alba* is another plant species for which AG inhibitory activity has been well-studied [44], and its leaf extracts exhibited AG inhibition activity with an IC_50_ value of 91.63 µg/mL compared to that of acarbose (IC_50_: 402.33 µg/mL) [45]. A range of bioactive compounds isolated from the leaves of Mango (*Mangifera indica* L.) were screened for their inhibitory effect against AG and among them arjunolic acid and actinidic acid exhibited inhibitory activity with IC_50_ values of 239.60 ± 25.00 and 297.37 ± 8.12 μM, respectively [46]. Similarly, extracts from the peel of fruits of Jackfruit (*Artocarpus heterophyllus*) displayed the strongest inhibitory activity (IC_50_: 0.05 mg/mL) followed by its seed (IC_50_: 1.79 ± 0.15 mg/mL), pulp (IC_50_: 6.81 ± 0.52 mg/mL), and flake (IC_50_: 10.52 ± 0.73 mg/mL) extracts [47]. Grape seed and green tea extracts were also found to have strong inhibitory activity against AG with an IC_50_ values of 1.2 ± 0.2 and 0.5 ± 0.1 µg/mL, respectively. These inhibition potencies were much stronger than the inhibitory effect obtained from acarbose (IC_50_: 91.0 ± 10.8 µg/mL) [48].

## 4. Common Vegetables with AG Inhibitory Activities

Major vegetables belonging to the Solanaceae family, such as pepper, tomato, eggplant, and potato, have been studied for their inhibitory activity against AG enzymes. Water extracts from the fruits of several pepper lines were examined for their effects on AG enzymes and the inhibitory percentages from a red sweet pepper variety were found to be 57% and 48% against yeast and rat AG enzymes, respectively [49]. Similarly, ethanol and water crude extracts from fruits of some pepper cultivars consumed in Korea exhibited close to full inhibitory activity against yeast AGs, compared to acarbose that showed 50% inhibition at 25 mM [50]. Luteolin-7-*O*-glucoside flavonoid isolated from pepper leaves showed a similar level of inhibitory activity (IC_50_: 15 µM) compared to acarbose [51]. Recently, methanolic extracts of several potato tubers exhibited AG inhibition with IC_50_ values ranging from 42.42 ± 0.94 to 78.65 ± 0.48 µg/mL, which is less potent than acarbose (IC_50_: 15.65 µg/mL) [52]. Fruit extracts from two eggplant species *S*. *macrocarpon* and *S*. *melongena* exhibited a moderate inhibitory effect with IC_50_ values of 71.77 ± 0.50 and 63.24 ± 0.30 µg/mL, respectively [53]. Tomato leaf extracts were found to have much milder inhibitory activity (IC_50_: 1.14 to 6.48 mg/mL) against AGs than that of acarbose (IC_50_: 356 ± 20.6 µg/mL) [54]. The inhibitory potential of onions together with different vegetables against AGs was also investigated. The ethanol extract of onion powder displayed an average of 74.0% inhibition compared to bitter melon (36.7%), yam (27.2%), and pumpkin (25.2%) [55]. Ethyl acetate extracts obtained from shallot (*Allium cepa ascalonicum*) peels, peeled bulbs, and bulbs (the whole bulbs) exhibited strong AG inhibitory activity with an IC_50_ values of 0.012 ± 0.002, 0.035 ± 0.01, and 0.052 ± 0.01 mg/mL, respectively. In the study, the inhibitory activity obtained from the peel was found to be the highest compared to those from different parts of 25 different plant species, including *Rheum palmatum* roots (IC_50_: 0.016 ± 0.0002 mg/mL), *Cinnamomum zeylanicum* bark (IC_50_: 0.018 ± 0.0006 mg/mL), *Brassica juncea* leaves (IC_50_: 0.21 ± 0.02 mg/mL), *Capsicum frutescens* fruits (IC_50_: 2.12 ± 0.4 mg/mL), *Allium sativum* bulbs (IC_50_: 2.51 ± 0.5 mg/mL), *Actinidia deliciosa* peels (IC_50_: 2.77 ± 2.4 mg/mL), and *Glycine max* beans (IC_50_: 12.83 ± 4.0 mg/mL) [56].

Natural acylated anthocyanins extracted from *Ipomoea batatas* showed strong maltase inhibitory activity with an IC_50_ value of 0.36 mg/mL [57]. Similarly, red cabbage varieties exhibited increased AGI activity, with total highest phenolic and diacylated anthocyanin activity obtained from the Koda variety (with an IC_50_ value of 3.87 ± 0.12 mg/mL) [58]. Aqueous radish sprout extract was also reported to cause 50% reduction of AG activity at the concentration of 60.7 ± 1.2 mg/mL [59]. Lactucaxanthin, a carotenoid extracted from lettuce, showed AG inhibition with an IC_50_ value of 1.84 mg/mL, while acarbose showed a value of 16.19 μg/mL [60]. Cucurbits are another major vegetable crops that possess inhibitory activity against AG enzymes. Bitter melon has been proved for hypoglycemic effects. Notably, protein extract from two genotypes of bitter melon (*Momordica charantia* var. *charantia* and *M. charantia* var. *muricata*) displayed 68.8% and 69.2% inhibition on AG activity, respectively. In the study, the IC_50_ values of *M. charantia* var. *charantia* (0.298 ± 0.034 mg/mL) and *M. charantia* var. *muricata* (0.292 ± 0.022 mg/mL) were not significantly different from the IC_50_ value of acarbose (0.28 ± 0.019 mg/mL) [61]. Likewise, methanol extracts from bitter melon fruits exhibited 50% inhibition on sucrase activity [62] and ethyl acetate extracts of *M*. *charantia* showed the highest AG inhibition activity (66.64% ± 2.94%) compared to *Trichosanthes cucumerina* (Snake gourd: 61.91% ± 1.96*%), Lagenaria siceraria* (56.04% ± 1.72%), *Sechium edule* (51.49% ± 2.13%), *Benincasa hispida* (48.73% ± 0.98*%*), *Luffa acutangula* (43.93% ± 1.28%), and *Cucurbita maxima* (22.11% ± 0.90%) [63]. Extracts from different parts of yellow fleshed watermelon also reduced the activity of AG enzyme; the highest inhibitory activity was obtained from 70% ethanol extract of the leaf (IC_50_: 26.26 ± 0.29 μg/mL), followed by the seed (IC_50_: 32.50 ± 0.36 μg/mL), the flesh (IC_50_: 41.38 ± 1.04 μg/mL), and the rind (IC_50_: 45.44 ± 0.18 μg/mL) [64]. The aqueous extracts from okra (*Abelmoschus esculentus*) peels and seeds demonstrated inhibitory effect against glucosidase enzymes with an IC_50_ value of 142.69 ± 0.32 and 150.47 ± 0.28 μg/mL, respectively [65].

## 5. Analyses of AGI Activities in Plants

**Figure 3:**
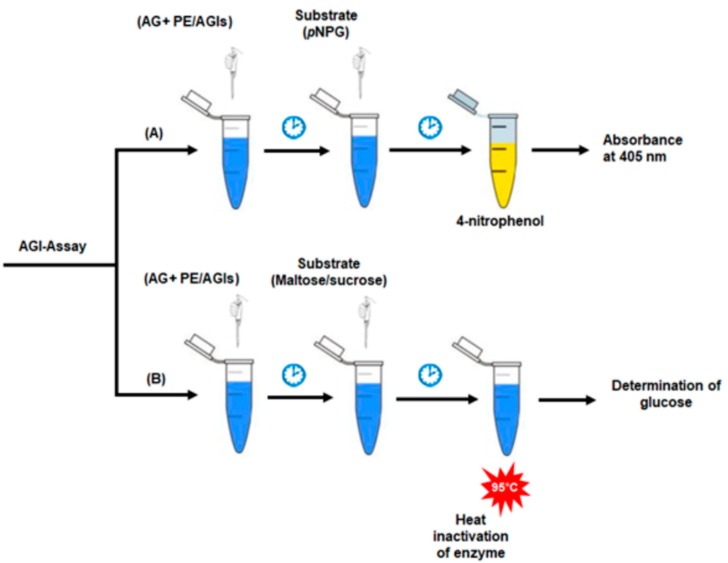
Schematic diagram of the procedure through which AGI activity is measured in plant extracts using different substrates; *p*NPG (**A**) and maltose/sucrose (**B**). *p*NPG, *p*-nitrophenyl-α-d-glucopyranoside, AG, α-glucosidase; PE, plant extract; AGI, α-glucosidase inhibitor.

Generally, AGI analysis is an enzymatic assay that follows the same basic principles as most enzymatic assays. Mostly, measurement of AG inhibitory activity is based on colorimetric methods. *p*-nitrophenyl-α-d-glucopyranoside (*p*NPG) is a synthetic substrate that is hydrolyzed specifically by AGs into a yellow colour product (4-nitrophenol) that is usually quantified at 405 nm. Hence, measuring the amount of 4-nitrophenol produced from *p*NPG in the presence or absence of inhibitors is used to measure the inhibitory activity of plant compounds against AGs (Figure 3A; [66]). Similarly, several studies have used maltose or sucrose as a substrate to screen the inhibitory potential of plant extracts against AG enzymes based on the amount of glucose produced in the presence or absence of the inhibitors (Figure 3B; [67,68,69]). However, even though the measurement methods look similar, there is inconsistency in the choice of enzyme sources, concentration of enzymes and substrates, and sometimes incubation time, which result in variations in absolute values. Therefore, well-known AGI compounds are usually used as controls for comparison of the inhibitory potency of other inhibitors against AG enzymes. However, these inhibitors usually exhibit different inhibitory activities based on the origin of glucosidase enzymes. The representative AG inhibitors such as acarbose and glucono-1, 5-lactone inhibited enzymes obtained from rat, rabbit, and pig intestine but had no inhibitory effects on baker’s yeast AG enzyme. On the contrary, (+)-catechin exhibited good inhibition against yeast AG without significant inhibitory effects on the mammalian enzymes [67]. Moreover, acarbose and voglibose showed no inhibition against AG from yeast and *Bacillus stearothermophilus,* but they inhibited porcine small intestinal AG with IC_50_ values of 35.00 and 0.035 μg/mL, respectively [66]. However, acarbose has been used as a positive control for modest inhibition against the yeast AG enzyme activity in several cases. Of note, there are independent reports demonstrating that acarbose exhibited 50% inhibition of yeast enzyme activity at the concentration of 177.47 ± 6.28 μg/mL [70] and 200 μg/mL [71]. Therefore, apart from the inherent difference in inhibitor’s affinity toward different AG enzymes, other conditions in assays could also cause inconsistency of inhibitors’ potency. For example, concentrations of enzymes and substrates were reported to affect IC_50_ values of particular competitive inhibitors. Thus, determination of an inhibition constant (Ki) as a measure of absolute binding affinity would solve this matter as it is not affected either by substrate or enzyme concentrations [72]. In addition, the concentration of substrates should be around their Km values toward an enzyme in order to screen all types of inhibitors, such as competitive, non-competitive, and mixed type inhibitors [73].

## 6. Production of Vegetables with Higher AGI Activity

Very few vegetables have been released/commercialized with approval of ingredients to help in managing type-2 diabetes. “Dangjo” and “Wongi No. 1” are two well-known Korean pepper varieties that have been specifically developed for higher AGI activity [51,74]. Since a range of bioactive compounds have been reported to possess AG inhibitory activity, as described above, increasing the amount of such inhibitors in vegetables can be a critical step for the production of vegetables with higher AGI activity. Most AGI activities are associated with the genetic architecture of quantitative traits (secondary metabolites; [75]). Thus, conventional breeding methods based on selection or hybridization with the aid of chemical analytical tools can also be used to produce vegetables with increased amounts of AGIs. It has been reported that various vegetables contain bioactive metabolic compounds (Table 2) and varieties/cultivars with higher content of AG inhibitory compounds from each species can be also generated through molecular breeding and/or biotechnological approaches. Genetic analyses of gene interactions and heritability for these compounds are necessary to establish appropriate breeding programs. However, few studies have reported the genetic parameters linked to the quantitative analyses of secondary metabolites including phenolic acids, flavonoids, alkaloids, and terpenes. A recent report of moderate to high heritability values for chlorogenic acid content in eggplants indicates that selection for these traits is likely to be a stepping stone for launching efficient breeding programs targeting production of vegetables with improved content of bioactive compounds [76].

Recent advances in molecular biology and genomics have given new insight into the biosynthesis pathways of secondary metabolites, including phenolic acids and flavonoids, and allowed the identification of quantitative trait loci (QTLs) involved in the pathways. A number of metabolic quantitative trait loci (mQTLs) and candidate genes responsible for the synthesis of phenolic acids were identified in a population generated from an interspecific cross between *S. lycopersicum* and *S. Chmielowski* [85]. Based on the knowledge gained by genetic/genomic approaches, vegetables with an increased amount of such compounds via enhanced biosynthesis can be produced through genetic transformation. For instance, co-introduction of the *PRODUCTION OF ANTHOCYANIN PIGMENT1* (*PAP1*), a regulatory gene from *Arabidopsis,* and the *CHALCONE ISOMERASE* (*CHI*) gene, from *Allium cepa,* into tomato caused 130- and 30-times higher levels of rutin (a bioactive flavonol) and total anthocyanin content, respectively, than those found in wild tomato skin [86].

## 7. Future Perspectives

**Figure 4:**
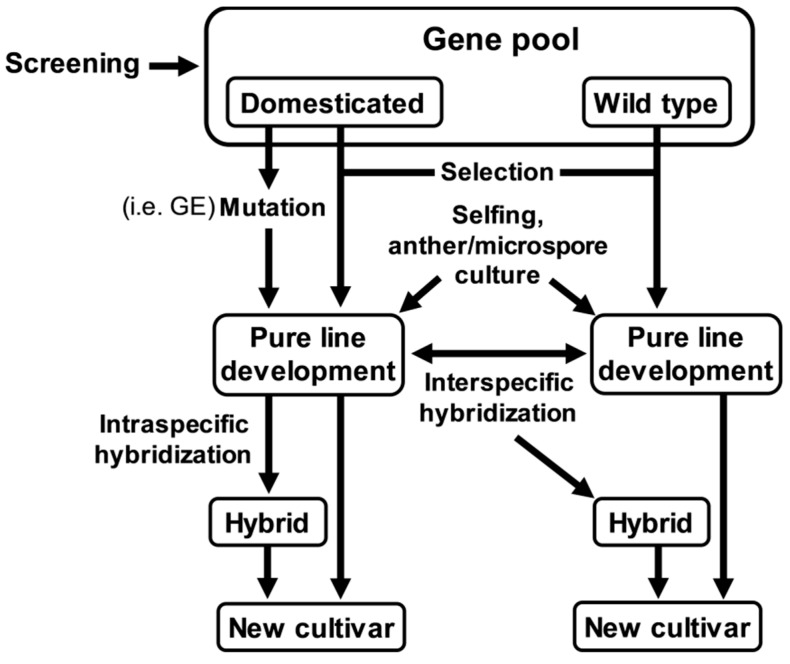
A flowchart depicting breeding strategies for developing varieties with high AGI content. Genome editing (GE) can be used for creating targeted gene modifications as a method of mutation breeding. Anther/microspore culture technology can also contribute to reduce the time required to develop pure lines. Novel genes can be introgressed into the cultivated species through interspecific hybridization with the wild species.

Diverse groups of secondary metabolites exist in plants and the current technologies in analytical chemistry as well as biochemistry provide opportunities to develop efficient high-throughput screening methods for compounds with AGI activity. Considerable effort has also been put into producing common vegetables containing higher amounts of AG inhibitory compounds. In addition, the content of AGIs in vegetables can be increased by controlling environmental factors for plant growth and development, such as temperature, light (i.e., period and quality), nutrients, water availability, and so on. A generation of new varieties with higher AG inhibitory compound content is also attainable through breeding with the aid of molecular genetic technologies. Current advances in molecular genetic/genomic technologies, followed by the availability of plant genomic and/or metabolomic information, has made it possible to acquire precise profiles of AGI candidates and even to identify the genes responsible for their biosynthesis. Genome editing (GE) technologies including CRISPR/Cas9 systems are also likely to be utilized for enhanced production of AG inhibitory compounds in common vegetables (Figure 4). We believe that production of vegetables with higher AGI activity would be of great help in treatment/management of type-2 diabetes, since vegetables are rich in fiber and/or are high in nitrates, and will generally support improved levels of healthy cholesterol and lower blood pressure. Additionally, adverse effects of AGIs, such as flatulence, abdominal discomfort, and diarrhea, can be reduced since they are generally dependent on AGIs dosage and duration of therapy/treatment. However, the goal—production of common vegetables with desirable AG inhibitory compound content—should be achieved without compromising crop yield, quality, and customers’ preference.

## Figures and Tables

**Table 1:** Classes of natural α-glucosidase inhibitor compounds and their IC_50_ (Half-maximal inhibitory concentration) values.

Classes of Compound	Chemical Structure	IC_50_ Value	Reference
**Alkaloids**			
Vasicine	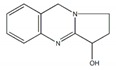	125 µM (0.8 µM)	[24]
Piperumbellactam B	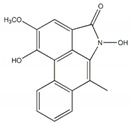	43.8 µM (426 µM)	[25]
Piperumbellactam C	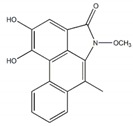	29.6 µM (426 µM)	
**Flavonoids**			
Quercetin	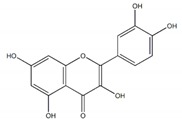	7 µM	[26]
Luteolin	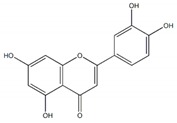	21 µM	
Cyanidin	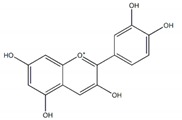	4 µM	
Baicalein	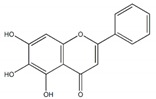	0.26 µM (0.02 µM)^a^	[27]
Quercitrin (quercetin-3-*O*-α-l-rhamnopyranoside	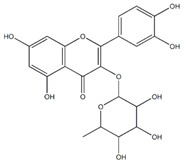	~0.5 mM (0.90 mM)	[28]
Isoquercetin	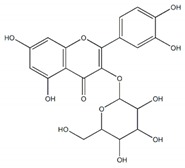	64.1 ± 3.3 µM (1.50 ± 0.14 µM) Maltase 42.5 ± 1.2 µM (2.38 ± 0.02 µM) Sucrase	[29]
Cyanidin-diglucoside	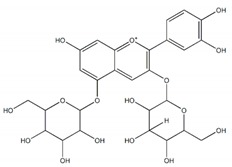	14.7 µg/mL	[30]
Pelargonidin-3-rutinoside	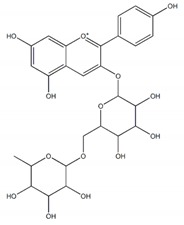	64.5 µg/mL	
Epicatechin-(4β,8)-Epicatechin gallate	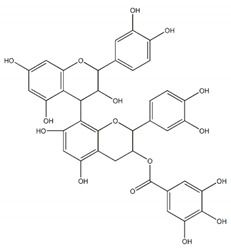	0.31 µM (5.3 µM)	[31]
Epicatechingallate	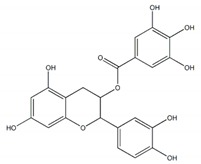	0.71 µM (5.3 µM)	
**Terpense**			
22α-hydroxychiisanoside	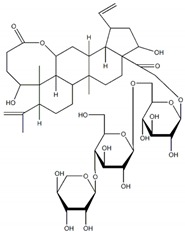	819.7 µM (788.6 µM)	[32]
7β-acetoxy-6β-hydroxyroyleanone	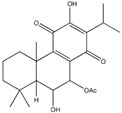	108.2 µM (131.2 µM)	[33]
Spicatanol	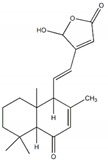	34.1 µM (23.8 µM)	[34]
Lupeol	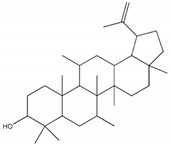	7.18 µg/mL (9.68 µg/mL)	[35]
**Phenols**			
*p-*hydroxycinnamic acid	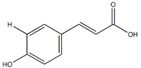	90.8 µg/mL (230.4 µg/mL)	[36]
Protocatechuic acid	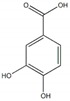	85.1 µg/mL (230.4 µg/mL)	
Trans-*N*-(p-Coumaroyl)tyramine	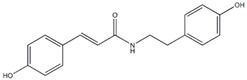	4.47 µM (168.95 µM)	[37]
2,4-dimethoxy-6,7-dihydroxyphenanthrene	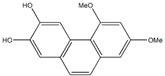	0.40 mM (3.52 mM)	[38]
Ferulic acid	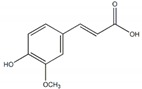	4.9 mM (1.7 mM)	[39]
Ellagic acid	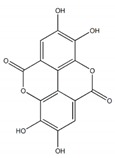	18.4 µg/mL	[30]
Umbelliferone	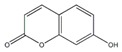	7.08 µg/mL (9.68 µg/mL)	[34]
**Iminosugars**			
*N*-(9′-methoxynonyl)-1-deoxynojirimycin	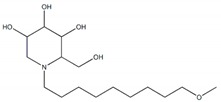	0.015 µM	[40]
*N*-(6′-4″-azido-2″-nitrophenylamino) hexyl-1-deoxynojirimycin	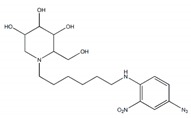	0.017 µM	

Values in parentheses are those of positive controls in references. ^a^ Concentration of a positive control showing 100% inhbition.

**Table 2:** Variability of total phenolic acid and total flavonoid content in several major vegetables.

Vegetables	Phenolic Acids (g·kg^−1^)	Flavonoids (g·kg^−1^)	Reference
Tomato	1.3–3.2	1.1–2.4	[77]
Pepper	7.95–26.15	4.64–12.84	[78]
Onion	3.43–22.19	0.0012–0.98	[79,80]
Garlic	3.4–10.8	0.1–0.22	[81,82]
Eggplant (*Solanum melongena*)	7.4–14.3	0.03–0.26 (fw)	[83,84]
